# Overview of Short Peptides for Hair Loss

**DOI:** 10.3390/biomedicines14040864

**Published:** 2026-04-09

**Authors:** Changxin Fan, Yanyu Chen, Qinghua Huang, Wai Ying Ou, Cancan Zhang, Yanlin Sun, Tianyue Wu, On Yee Leung, Hei Ching Iu, Jiacheng Shi

**Affiliations:** 1 Division of Life Science, The Hong Kong University of Science and Technology, Clearwater Bay, Kowloon, Hong Kong, China; 2 Department of Chemical and Biological Engineering, The Hong Kong University of Science and Technology, Clearwater Bay, Kowloon, Hong Kong, China; qhuangas@connect.ust.hk (Q.H.);; 3 Department of Microbiology, University of Georgia, Cedar Street Building C, Athens, GA 30602, USA; czhangda@connect.ust.hk; 4 Department of Physics, The Hong Kong University of Science and Technology, Clearwater Bay, Kowloon, Hong Kong, China; 5 Department of Computer Science and Engineering, The Hong Kong University of Science and Technology, Clearwater Bay, Kowloon, Hong Kong, China; 6 College of Life Science and Technology, Huazhong University of Science and Technology, Wuhan 430074, China; d202581294@hust.edu.cn

**Keywords:** biomedicines, short peptides, hair loss, alopecia, mechanisms, nanotechnology

## Abstract

Hair serves essential functions, including mechanical sensing, head protection, and body temperature regulation, while also playing a significant role in human aesthetics. However, factors such as hormonal imbalances, autoimmune disorders, infections, and psychological stress contribute to the widespread issue of hair loss, particularly among the elderly, adversely affecting self-confidence and self-esteem. Although treatments such as minoxidil, finasteride, and dutasteride have received regulatory approval, their associated side effects, such as sexual dysfunction, neuropsychiatric issues, and cardiovascular symptoms, can impede patient recovery. While follicular unit transplantation and stem cell therapy show promising outcomes, they are not suitable for all types of hair disorders. Short peptides that mimic intracellular signals and exhibit diverse biological effects have emerged as a promising approach for stimulating hair regrowth. By combining different formulations and nanosystems, the limitations of short peptides can be effectively addressed. This review systematically summarizes recent advances in peptide-based treatments for hair loss, highlighting their advantages and limitations.

## 1. Introduction

Hair is a natural component of the human body, hypothesized to have originated as mechanoreceptor units [1]. From primitive societies to modern humans, hair is believed to protect the head from ultraviolet radiation, maintain humidity, and regulate body temperature. Although humans can survive without hair, indicating that it does not serve vital functions, the psychological significance of hair appears to be profound. Hair plays important roles in social and sexual display, symbolizing femininity in women and masculinity in men [2]. Hairstyles are also crucial for human aesthetics, with practices such as perming and dyeing becoming popular fashion trends [3].

**Figure 1:**
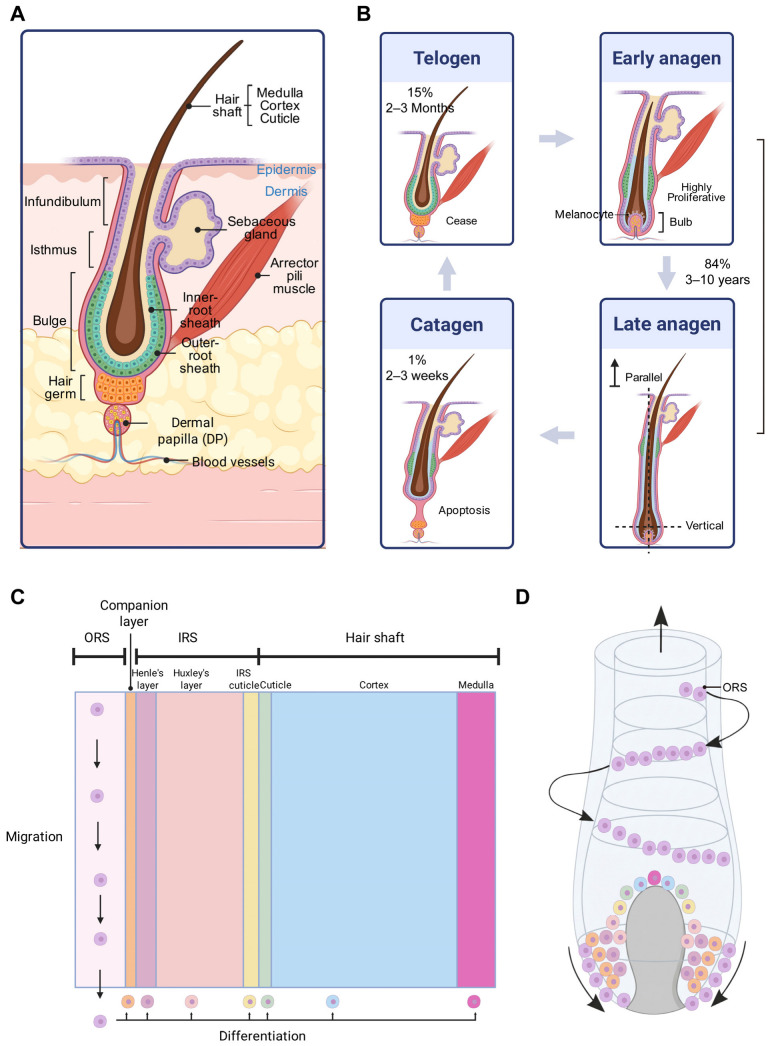
The anatomy and growth cycle of hair follicles. (**A**) An evidence-based diagram of the hair follicle structure during the telogen phase. The mesenchymal niche is primarily composed of a dense group of dermal fibroblasts known as the dermal papilla (DP). Cells in the hair germ are the first to express genes indicative of stem cell activation and are the first to proliferate at the onset of a new hair regeneration cycle [11,12]. The bulge is defined as the location of hair follicle stem cells (HFSCs), with Krt15^+^/integrin 6^+^ or CD34^+^/integrin 6^+^ bulge cells regarded as HFSCs [13,14]. The isthmus is the epithelial compartment, where isthmus cells exhibit stem cell characteristics and can generate HF lineages. Sebocytes, the specialized keratinocytes of the sebaceous gland (SG), secrete sebum, an oily substance that protects, lubricates, and waterproofs the hair [15]. Specialized sensory neurons innervate each HF, and the arrector pili muscle attaches to the side of the HF at the level of the bulge region. (**B**) Evidence-based diagrams of the HF growth cycle are shown. Approximately 84% of hairs are actively growing during the anagen phase, which lasts 3–10 years and determines hair length [5]. In the early anagen phase, the hair germ gradually envelops the DP to initiate bulb formation. This results in a layer of highly proliferative precursors that will differentiate into the various cell types of mature hair. (**C**) A conceptual illustration is exhibited. The differentiated cells collectively form seven concentric layers of the inner root sheath (IRS) and hair shaft [16,17]. The orange color represents the companion layer, while the light pink color denotes Henle’s layer. The light red color signifies Huxley’s layer, and the yellow color indicates the IRS cuticle. Additionally, the light green color corresponds to the cuticle, the light blue to the cortex, and the pink to the medulla. (**D**) An evidence-based diagram is exhibited. The latest experimental evidence indicates that outer root sheath (ORS) cells exhibit a spiraling downward movement into the lower bulb region. This dynamic motion generates a pulling force that facilitates the extrusion of hair fibers [17]. (**B**) About 1% of hair follicles transition to the catagen phase, which lasts 2 to 3 weeks, during which most follicular keratinocytes undergo apoptosis, and follicular melanogenesis ceases. The bulge region becomes disconnected from the DP. Approximately 15% of hairs enter the telogen phase, during which they remain inactive at the hair bulb for 2 to 3 months before reentering the anagen stage, thus repeating the cycle. Eventually, hair is shed from the follicle. Created in BioRender. Fan, C. (2026) https://BioRender.com/wzhitd0, https://BioRender.com/0qjircd, and https://BioRender.com/i4onsly.

Hair grows from hair follicles (HFs), which are self-contained mini-organisms within mammalian skin. Hair follicles undergo cyclical phases to elongate hair, which can be divided into three stages: the anagen phase, catagen phase, and telogen phase (Figure 1). Hair loss can be categorized into four types based on etiology, including hormonal disorders, such as androgenetic alopecia (AGA) [4], immunological disorders, such as alopecia areata (AA) [5,6], infections, such as tinea capitis [7], and stress-induced alopecia, exemplified by telogen effluvium (TE) [5,8,9]. The demand for treatments that alter hair growth and appearance has led to a multibillion-dollar industry [9]. Topical minoxidil and oral finasteride are widely used. However, their effects are variable and generally observed in only a minority of patients, making it challenging to predict treatment efficacy on an individual basis [9]. Physical therapies, including hair transplantation, microneedling, low-level laser therapy (LLLT), and acupuncture, also have limitations [5]. The reasons include limited availability of hair follicle donor sites, limited accessibility to specialized equipment, and lengthy treatment duration, which may not be convenient for individuals. Stem cell therapy is not yet widely commercialized. Dietary supplementation with natural plant extracts, controlled shampoo usage, and lifestyle modifications are widely accepted nonmedical strategies for preventing hair loss. However, these non-medical treatments may not yield satisfactory outcomes for individuals with severe hair loss.

Short peptides, which exhibit high accessibility and diverse effects, hold promise. A phase 2A trial of PP405, a peptide that activates hair follicle stem cells, demonstrated statistically significant hair regrowth in just eight weeks among 78 patients, showcasing a rapid onset compared to traditional therapies [10].

With multifaceted mechanisms of action, superior biocompatibility, and the potential for versatile combination therapies, peptides represent a promising emerging approach. However, concerns remain regarding the shortage of clinical trials, constraints of model systems, and biological heterogeneity. This paper evaluates the advantages and limitations of peptides for the treatment of hair loss.

## 2. Hair Loss Is a Historical Problem of Human Beings

### 2.1. Brief History of Peptides for the Treatment of Hair Loss

**Figure 2:**
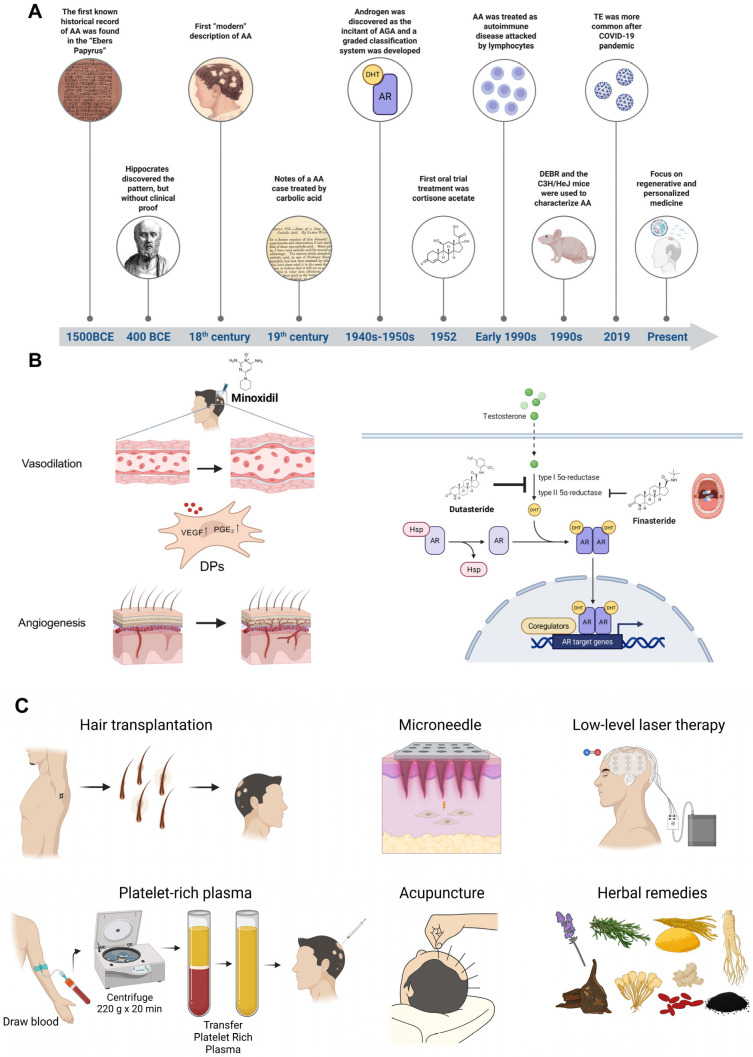
Hair loss research history and existing therapies (**A**) Evidence-based diagrams of some significant time points in hair loss research history are exhibited [26,27,28]. (**B**) Conceptual illustrations are exhibited. The FDA approves Minoxidil for treating hair loss in both men and women. While the precise mechanism of action remains unclear, it is hypothesized to involve vasodilation, upregulation of vascular endothelial growth factor and prostaglandin PGE_2_ secreted by DPs, and promotion of angiogenesis [29,30,31,32]. Finasteride selectively inhibits type II 5α-reductase, reducing the conversion of testosterone to dihydrotestosterone (DHT). In contrast, dutasteride inhibits both type I and type II isoforms of 5α-reductase. (**C**) Evidence-based diagrams of physical therapies and traditional Chinese medicine for hair loss treatment are shown. Hair transplantation involves relocating hair follicles from donor sites on the patient’s body to regions affected by hair loss. Microneedling creates controlled micro-injuries that stimulate HFSC activation and regeneration. Low-level laser therapy (LLLT) promotes hair growth by releasing nitric oxide, enhancing cellular metabolism, and blood flow. Platelet-rich plasma (PRP) therapy delivers a high concentration of growth factors to the scalp, stimulating follicular activity. Acupuncture may improve scalp circulation and modulate physiological pathways associated with hair growth. Traditional Chinese herbal remedies, such as lavender, rosemary, rice bran extract, ginseng, *Polygonum multiflorum*, *Angelica sinensis*, ginger, matrimony vine, and black sesame, have been used to promote hair growth through various pharmacological mechanisms. Created in BioRender. Fan, C. (2026) https://BioRender.com/7xpc8db, https://BioRender.com/rauluo0, and https://BioRender.com/1hfr63k.

Concerns about hair loss, particularly alopecia areata (AA), have persisted for over three millennia [18,19]. This enduring concern reflects the psychological impact of hair loss, despite its non-lethal nature (Figure 2A).

The foundation of peptide-based hair therapeutics dates back to 1973, when Loren Pickart first identified the tissue-regenerative properties of the copper-binding tripeptide GHK-Cu (glycyl-L-histidyl-L-lysine) [18,20,21]. In 1984, the successful isolation and culture of human dermal papilla cells (hDPCs) *in vitro* addressed the challenge of obtaining sufficient human tissue [22]. This breakthrough catalyzed further research, culminating in a landmark in 2007. Pyo et al. demonstrated that a related analog, AHK-Cu, stimulated the elongation of human hair follicles, firmly establishing the role of copper peptides in *ex vivo* research [23].

In 2004, a pivotal study by Philp et al. showed that thymosin β4 activated HFSCs, promoting hair growth [24]. The 2010s saw a surge in commercial innovation, with clinical data validating synthetic peptide complexes as viable topical alternatives for hair repair. For instance, Capixyl^TM^ features Ac-KGHK, which was developed to mimic natural matrilines. It stimulated the production of extracellular matrix (ECM) proteins and reduced the production of pro-inflammatory cytokines [25].

A distinct increase in peer-reviewed publications on peptide treatments began in 2019. It was driven by advancements in computational bioinformatics and a clinical need for non-hormonal therapies to address telogen effluvium (TE). This trajectory highlights a definitive shift away from 5α-reductase inhibitors toward multi-peptide regenerative combinations.

### 2.2. Hair Loss Has Multiple Etiologies

#### 2.2.1. Hormonal Imbalances

The most common form of hormonally driven hair loss is hereditary AGA, also known as patterned hair loss (Table 1). A large-scale study in South Asia found that approximately 14.1% of the male and 5.6% of the female population are affected by AGA [33,34,35]. It is characterized by diffuse thinning over the crown and frontal scalp, with preservation of the frontal hairline [36]. The proposed pathogenic mechanism involves increased androgen receptor (AR) expression in scalp hair follicle (HF) cells, resulting in hypersensitivity to testosterone derivatives [37]. Although the precise mechanism remains unclear, mutations near the AR regulatory region have been implicated in HF miniaturization [38].

#### 2.2.2. Autoimmune Disorders

Alopecia areata (AA) is the most prevalent autoimmune form of hair loss, affecting both sexes equally. In Asian populations, 85.5% of cases occur before the age of 40 [49]. The primary etiology of AA is genetic predisposition. Specific alleles have been identified as susceptibility markers, including mutations in the human leukocyte antigen (HLA) alleles [50]. In addition to genetic factors, environmental triggers such as certain viral infections can exacerbate AA by increasing the production of pro-inflammatory cytokines, which eventually attract CD8^+^ immune cells. This undermines the immune privilege of the anagen-phase HF and ultimately leads to hair loss [51].

#### 2.2.3. Infections

Infections are also a prominent cause of alopecia. Tinea capitis is a common scalp fungal infection primarily affecting children. It is most frequently caused by dermatophyte fungi such as *Trichophyton tonsurans* and *Microsporum canis* [7]. These fungi produce a range of proteolytic enzymes that function over a broad pH range, facilitating hair shaft penetration [52]. Clinical presentations vary depending on the infecting species but commonly include gray patches, black dots, and diffuse alopecia. In severe cases, inflammation or kerion lesions may develop [53].

#### 2.2.4. Stress-Induced Alopecia

The most prevalent form of stress-induced alopecia is telogen effluvium (TE), first described in 1961 as a non-scarring, diffuse hair shedding. TE is typically a delayed response to premature anagen termination, leading to early entry into catagen and telogen. This process is often triggered by metabolic stress resulting from either emotional or physical trauma [8]. Stressors may disrupt key regulatory molecules in the HF cycle, such as fibroblast growth factor (FGF) and transforming growth factor-β (TGF-β), promoting a shift from anagen to catagen [54].

### 2.3. Present Medications for Hair Loss Have Notable Limitations

The pharmacological treatment of hair loss, particularly AGA, primarily relies on two FDA-approved medications: topical minoxidil and oral finasteride, as well as the off-label use of dutasteride (Table 2). Although these drugs are considered first-line therapies, each presents notable limitations.

Minoxidil is available as a topical solution or foam. It promotes hair growth by prolonging the anagen phase, shortening the telogen phase, and enlarging miniaturized hair follicles (Figure 2B) [29,30]. Common adverse effects include irritant contact dermatitis, scaling, and itching, often attributed to the propylene glycol component, which can lead to poor patient compliance. Additionally, hypertrichosis, or undesired body hair growth, may occur. The efficacy of minoxidil depends on sulfotransferase enzyme activity within hair follicles; individuals with low enzymatic activity are typically non-responsive. Perhaps the most significant limitation is the requirement for continuous use; discontinuation often leads to hair loss resuming within 12 to 24 weeks [32].

Finasteride and dutasteride are oral 5α-reductase inhibitors (5ARIs) (Figure 2B) [55,56,57,58]. Despite their effectiveness, both drugs share similar limitations. First, while 5ARI therapy may reduce the incidence of low-grade prostate cancer, studies have suggested a possible increased risk of high-grade prostate cancer [59,60]. Second, there is concern regarding the potential for mood-related side effects. Some studies have reported an association between 5ARI usage and depressive symptoms, though a definitive causal relationship has not been established [61]. Third, sexual dysfunction is the most commonly reported adverse effect of 5ARI treatment, including erectile dysfunction, ejaculatory disorders, and decreased libido [62]. These side effects are often dose-dependent and may resolve upon discontinuation [63].

### 2.4. Limitations of Commercial and Clinical Therapies

In addition to small-molecule therapeutics, physical and biological approaches for treating hair loss have gained increasing prominence (Table 3). One of the most common solutions, hair transplantation, involves reallocating healthy HFs from donor areas of the patient’s body to regions affected by hair loss (Figure 2C) [68]. However, this procedure primarily serves patients with severe alopecia, and its success heavily depends on the availability of donor hair and the survival of transplanted follicles during and after surgery [5,68,69].

Microneedling creates controlled microinjuries in the scalp, potentially activating HFSCs and initiating the transition to the anagen phase [70]. Similarly, low-level laser therapy (LLLT) uses energy-carrying electromagnetic waves, typically in the red or near-infrared wavelengths, to stimulate nitric oxide release, which activates signaling pathways that promote cell mitosis and proliferation [5,71,72]. Commercial devices, including laser combs and caps utilizing this mechanism, are widely available [71]. These methods are often combined with other topical medications, as they can enhance the transdermal delivery of topical compounds [70]. However, the effectiveness of these treatments is highly dependent on the precise control of key parameters, such as microneedle length and density [70,73,74], or LLLT wavelength, intensity, and duration [72,75], which can vary significantly between individuals and types of hair loss.

Another emerging method is platelet-rich plasma (PRP) therapy, which involves extracting and concentrating platelets from the patient’s blood and injecting them into areas of thinning hair or administering them alongside other treatments (Figure 2C) [76,77,78,79]. However, the exact mechanisms of action remain unclear, resulting in a lack of standardized protocols for optimal efficacy [76,78,79].

Traditional Chinese medicine (TCM) is particularly prevalent in many Asian countries, where it is reported to be effective in alleviating hair loss symptoms [80]. Common treatments include oral or topical herbal remedies, extracts, and essential oils derived from medicinal plants such as rosemary and Korean red ginseng [81,82,83]. Their effects are thought to involve hormonal regulation through neural pathways and/or modulation of immune responses [80]. However, treatment outcomes heavily depend on the practitioner’s expertise and can vary significantly from patient to patient. The therapeutic effects are often modest, particularly in the short term, indicating a need for new solutions.

**Table 3:** Limitations of current therapies.

Approved Method	Key Limitations	References
Hair transplantation	Limited donor hair supply and graft survival; restricted achievable hair density; prolonged recovery period; procedure-related trauma and potential scarring.	[68,69]
Microneedling	Efficacy is highly dependent on precise control of device parameters (depth, frequency, technique); the procedure can be painful and may require topical anesthesia.	[70,73,74]
Low-level light therapy (LLLT)	Clinical response is sensitive to specific treatment parameters (wavelength, dose, duration); overall effectiveness may be limited and variable between patients.	[72,75]
Platelet-rich plasma (PRP)	Mechanisms of action are incompletely understood; it is often used as an adjunct rather than as a standalone therapy; the injection can cause discomfort and requires repeated sessions.	[76,77]
Acupuncture/moxibustion	Requires prolonged treatment courses; biological mechanisms remain poorly defined; outcomes are operator-dependent and may vary substantially between practitioners and patients.	[80,84,85]
Herbal remedies	Mechanisms of action are not well established; evidence for effectiveness is limited and inconsistent; product quality and standardization can vary.	[81,82]

## 3. The Classifications of Short Peptides for Hair Loss

Peptides facilitate hair follicle regeneration, extend the anagen phase, and prevent hair loss through various sources and mechanisms. This section categorizes peptides by their primary molecular mechanisms, providing a systematic framework that aligns with current understanding of hair follicle biology.

### 3.1. Peptides Activating the Wnt/β-Catenin Signaling Pathway

**Figure 3:**
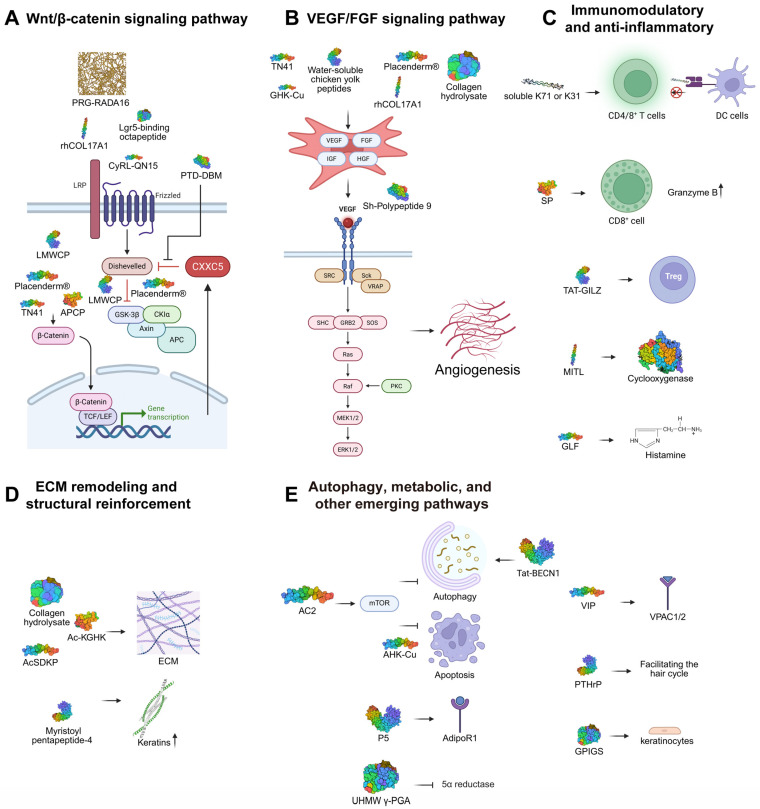
Common mechanisms of peptides in hair loss treatment (**A**) An evidence-based diagram is exhibited. Multiple peptides, including Placenderm^®^, APCP, TN41, and LMWCP, activate the Wnt/β-catanin pathway by inhibiting GSK-3β or stabilizing β-catenin. CyRL-QN15 binds to the Frizzled-7 receptor, a classic receptor for Wnt proteins, while an Lgr5-binding octapeptide interacts with LGR5, a key component of the canonical Wnt signaling pathway. PRG-RADA16 and rhCOL17A1 also activate this pathway, although their mechanisms remain unclear. PTD-DBM disrupts the CXXC5-Dvl interaction, as CXXC5 is a negative regulator of the Wnt/β-catenin pathway. (**B**) A conceptual illustration is exhibited. VEGF and other growth factors are upregulated by GHK-Cu, TN41, Placenderm^®^, rhCOL17A1, CH, and water-soluble chicken yolk peptides, which activate the VEGF signaling pathway in endothelial cells, thereby promoting angiogenesis and enhancing blood supply. Sh-Polypeptide 9 mimics VEGF by directly binding to VEGF receptors. (**C**) An evidence-based diagram is exhibited. Soluble K71 or K31 can serve as a vaccine. SP stimulates granzyme B expression in CD8^+^ cells, leading to hair follicle regression. TAT-GILZ stimulates the function of Tregs. MITL stimulates cyclooxygenase activity, leading to PGE_2_ production, which suppresses alopecia induced by etoposide in neonatal rats. The anti-alopecia effect of GLF is mediated by histamine release in the same model as MITL. (**D**) An evidence-based diagram is exhibited. Ac-KGHK promotes the production of collagen III, collagen VII, and laminin. Collagen hydrolysate upregulates the expression of ECM production-related factors, including Elastin and HAS2. Myristoyl pentapeptide-4 stimulates keratin expression. (**E**) An evidence-based diagram is exhibited. AC2 suppresses autophagy and apoptosis through the mTORC1 signaling pathway. Tat-BECN1 can induce autophagy, thereby delaying the onset of AA. P5 binds AdipoR1 to activate the AMPK signaling pathway. And UHMW γ-PGA inhibits the activity of 5α-reductase. VIP binds the VPAC receptors to prevent the collapse of immune privilege. PTHrP was hypothesized to facilitate the hair cycle. GPIGS promotes the proliferation of hair keratinocytes. Created in BioRender. Fan, C. (2026) https://BioRender.com/42m8u60.

The Wnt/β-catenin signaling pathway is a key for stem cell activation, HF morphogenesis, and growth induction. Abnormal Wnt signaling is related to AGA and age-related alopecia. Multiple peptides regulate this pathway by inhibiting GSK-3β and stabilizing β-catenin (Figure 3A) [86,87].

The oral administration of low molecular weight porcine placenta peptides (Placenderm^®^) in an AGA C57BL/6 mouse model accelerated hair regrowth. It prolonged the anagen phase and resulted in significant improvements in HF number, size, thickness, and luster. VEGF and IGF1 were found to be increased via Western blot (WB) after Placenderm^®^ treatment. RT-qRCP assessed the Wnt/β-catenin activation. The mRNA levels of pathway inhibitors, such as GSK-3β and DKK1, decreased. The mRNA level of β-catenin increased. However, WB only showed decreased DKK1 and increased Wnt7a/b. It didn’t directly measure β-catenin levels, which weakens the credibility. An amount of 200 mg/kg Placenderm^®^ can achieve an effect comparable to 1 mg/kg bicalutamide [88].

A significant portion of peptides used for hair restoration is derived from natural collagen. AP Collagen Peptide (APCP) is a well-studied collagen-derived peptide obtained from the enzymatic hydrolysis of fish skin collagen, such as that from golden threadfin bream (*Nemipterus virgatus*). Rich in glycine-proline-hydroxyproline tripeptides, APCP typically has a low molecular weight of approximately 3–5 kDa. It has been found to promote human HF (hHF) growth after 10 days of transplantation in BALB/c nude mice. APCP achieves these effects by increasing the protein levels of phosphorylated β-catenin (Ser552/Ser675) and total β-catenin in hDPCs. Immunofluorescence (IF) further confirmed the increase in β-catenin and VEGF in the hHFs organ culture model. A hair regeneration experiment was conducted in C57BL/6 mice. After 16 days of treatment, the hair growth area, hair thickness, anagen/telogen ratio, and HF number were significantly increased compared with the negative control. Regarding hair growth promotion, 900 mg/kg APCP can achieve an effect comparable to 1 mg/kg minoxidil [89,90].

Low-Molecular-Weight Collagen Peptides (LMWCP) similarly regulate the Wnt/β-catenin pathway. In hDPCs, one hour of LMWCP treatment increased the protein levels of p-Akt (Ser473) and phosphorylated GSK-3β (Ser9). The increased β-catenin was detected after 24 h of treatment in a dose-dependent manner. Immunofluorescence further confirmed the translocation of β-catenin to the nucleus. A patch assay was conducted to confirm the effect of LMWCP on new hair inductivity. Epidermis and dermis cells were treated with LMWCP and then injected into the hypodermis of BALB/c nude mice. After 2 weeks, the LMWCP-treated group had more new HFs than the negative control. LMWCP also increased the hair growth rate in the hHF organ culture model. Finally, the hair regeneration function of LMWCP was assessed in telogenic C57BL/6 mice. Wnt3a, β-catenin, PCNA, Cyclin D1, and VEGF increased in dorsal skin on day 13, as detected by WB. However, the hair growth-promoting effect of 615 mg/kg LMWCP was not as strong as that of 3% minoxidil [91]. The research methods and logic of LMWCP are quite similar to APCP. Both LMWCP and APCP can increase keratin expression in the dorsal skin of mice after treatment.

The recombinant type XVII collagen (rhCOL17A1) is derived from the α1 chain of human COLXVII. RT-qPCR of hDPCs after treatment showed decreased Bax and increased Bcl-2, VEGF, IGF-1, and FGF-7, leading to improved hDPC survival. WB of hDPCs showed increased Wnt3a, β-catenin (Ser675)/β-catenin, and LEF-1. The DKK-1 protein level was found to have decreased. It indicated activation of the Wnt/β-catenin signaling pathway. However, the total protein level of β-catenin didn’t show any big difference. What’s more, WB also showed increased SHH, SMO, GLI-1, C-myc, and Cyclin D1, which indicated activation of SHH/GLI signaling pathways. Immunofluorescence showed that rhCOL17A1 upregulated its own expression while slightly decreasing MMP-9 expression in hDPCs. In 5% testosterone propionate-treated C57BL/6 mouse model, rhCOL17A1 treatment increased the hair growth score and cell proliferation in the dorsal skin. No positive control was used, and 0.5% *w*/*v* rhCOL17A1 achieved the best effect [92]. Immunofluorescence further confirmed that the expression levels of Wnt3a, LEF-1, SMO, EGFR, and COLXVII were increased in the dorsal skin of mice after rhCOL17A1 treatment.

Short peptides also regulate the Wnt pathway at the receptor level. CyRL-QN15, a peptide isolated from frog skin secretions, directly binds to the Frizzled-7 receptor, a key upstream component of the classic Wnt signaling pathway. CyRL-QN15 promoted wound healing and accelerated hair regeneration in diabetic Kunming (KM) mice. Immunofluorescence indicated an increased Ki67-positive area in CD34^+^ and Lgr5^+^ HFSCs. EdU^+^ cells were increased in the dorsal skin of mice after treatment. A similar hair regeneration experiment was also conducted in diabetic C57BL/6 mice. CyRL-QN15 treatment increased the area of regenerated hair and the anagen/telogen (A/T) ratio. An amount of 100 nmol/L CyRL-QN15 achieved a better effect than 100 mmol/L minoxidil [93]. Immunofluorescence indicated the increased β-catenin and the downstream Cyclin D1. In vitro, CyRL-QN15 treatment increased HFSC proliferation. The protein level of β-catenin and its downstream targets, such as Cyclin D1, c-MYC, and PCNA, increased as assessed by WB. Combination treatment with the Wnt/β-catenin signaling pathway inhibitor DKK1 and the Frizzled-7 receptor-blocking agent Fz7-21 can significantly reduce β-catenin and its downstream protein levels. It indicates that the function of CyRL-QN15 relies on the Wnt/β-catenin signaling pathway.

Also, PTD-DBM is a peptide that disrupts CXXC5-Dishevelled (Dvl) interactions. Combination treatment with PTD-DBM and Wnt3a synergistically increased the protein level of β-catenin and alkaline phosphatase (ALP), a prominent dermal papilla marker in hDPCs. Cotreatment with PTD-DBM and VPA, a GSK-3β inhibitor, significantly upregulated β-catenin, ALP, PCNA, keratin 14, and Erk, promoting hair regrowth in C3H mice [94]. Topical administration of 2 mM PTD-DBM and 500 mM VPA was more effective than 100 mM minoxidil.

An Lgr5-binding octapeptide has been shown to increase β-catenin nuclear translocation, thereby promoting hDPC proliferation. The mRNA levels of β-catenin downstream genes, including Lef1, Cyclin D1, and Myc, were increased, as determined by RT-qPCR. Wnt-5a and Wnt-10b concentrations in the culture media were increased, as measured by ELISA. Regarding growth factors, the mRNA levels of FGF2, FGF7, HGF, and VEGFA were markedly increased by octapeptide treatment. WB confirmed the upregulated p-Akt. Except for hDPCs, the proliferation of human ORS cells, human germinal matrix cells, and hHFSCs was also stimulated by octapeptide treatment. No positive control was used, and 100 μM octapeptide achieved the best result [95].

Another interesting peptide, TN41, comprises an N-terminal fragment of AIMP1 and a cell-penetrating motif. Aminoacyl-tRNA synthetase-interacting multifunctional protein 1 (AIMP1) can be secreted from HFSCs by Wnt3a stimulation and has distinct extracellular functions. HF-specific knocking out of AIMP1 will cause hair regeneration defects. After TN41 treatment, the hair regrowth areas significantly increased. The function was also demonstrated in the C57BL/6 mouse model. An amount of 100 nM TN41 can promote hair growth comparably to 3% minoxidil [96]. TN41 increased β-catenin levels in hDPCs *in vitro*. LEF1, Ki67, and c-Myc were upregulated in the dorsal skin of mice after treatment. TN41 increased β-catenin and ALP levels in DPCs in a dose- and time-dependent manner. The mRNA levels of KGF, HGF, IGF, and VEGF were increased in DPCs by TN41, while the inhibitory genes were not. Furthermore, TN41 promoted the elongation of hHFs. The model proposed that Wnt3a promotes HFSCs secreting truncated AIMP1, which activates the β-catenin signaling and promotes growth factor synthesis in DPCs.

PRG-RADA16 is designed to create nanofiber scaffolds that mimic the microenvironment of HF. This peptide combines the PRG adhesive motif with the self-assembling RADA16 peptide to form a 3D hydrogel network. It supports skin-derived precursors (SKPs) that can differentiate into various cell types. It increases the expression of important DP signature genes, including Wnt, BMP, VCAN, BMP6, and Alx3 in SKPs, thereby creating a biomimetic niche for hair-inductive differentiation. Neonatal epidermal cells and SKPs were mixed with different peptide hydrogels or Matrigel. It was further implanted into excisional wounds in nude mice. New hair shafts were counted under a dissecting microscope after 3 weeks. PRG-RADA16 was even better than Matrigel in supporting *de novo* HF genesis. However, functional hair regeneration from this scaffold has not been demonstrated *in vivo* [97].

### 3.2. Pro-Angiogenic Peptides Targeting VEGF/FGF Signaling

Adequate blood supply to the DP is essential for sustaining the metabolically active anagen phase. Pro-angiogenic peptides enhance follicular vascularization by upregulating VEGF, FGF, and related growth factors (Figure 3B). This mechanism shares similarities with minoxidil, which is thought to act, at least in part, through VEGF-mediated vasodilation [98].

Copper-binding peptides enhance vascularization, facilitating hair regeneration. Copper peptide (GHK-Cu), a tripeptide composed of Gly-His-Lys with Cu^2+^, has been found to increase the production of VEGF in dermal fibroblasts. This function stimulates microvascular angiogenesis and collagen/GAG synthesis, thereby facilitating hair growth. The hair growth promotion effect was demonstrated in a clinical study of 45 AGA patients [18]. GHK-Cu additionally promotes ECM turnover through matrix metalloproteinase (MMP) activation and exhibits anti-inflammatory properties, conferring a broader multi-target profile than other pro-angiogenic peptides [21].

AHK-Cu (L-Ala-L-His-L-Lys-Cu^2+^) has also been shown to promote the growth of DPCs *in vitro* and the *ex vivo* elongation of human HFs, although its specific signaling mechanism is less well characterized than GHK-Cu [23]. AHK-Cu treatment increased the cell viability assessed by the MTT method. Annexin/PI double staining also confirmed fewer apoptotic DPCs. WB detected increased Bcl-2 and decreased Bax protein levels in DPCs. Decreased cleaved caspase-3 and PARP protein levels were further validated.

There are also natural and biomimetic pro-angiogenic peptides. Sh-Polypeptide 9, a VEGF-like biomimetic peptide, promoted endothelial tubulogenesis and VEGF production more effectively than minoxidil in an *in vitro* co-culture system of DPCs and microvascular endothelial cells [99]. An amount of 2.5 mg/mL Sh-Polypeptide 9 upregulated β-catenin levels and downregulated IL-1 and caspase-3 levels in DPCs.

Water-soluble chicken egg yolk peptides, but not egg white peptides, can stimulate the production of VEGF in DPCs. It didn’t affect mouse growth and promoted hair growth in C3H mice. Egg yolk peptide diet at 0.1% achieved a similar stimulation effect as 1% minoxidil [100]. Oral administration is an important advantage of egg yolk peptide. Furthermore, in a 76-subject clinical study, 250 mg/day of egg yolk peptide achieved significantly higher hair density than the same amount of placebo after 24 weeks of treatment.

### 3.3. Immunomodulatory and Anti-Inflammatory Peptides

Peptides involved in immune response modulation can also aid in hair loss regeneration (Figure 3C). K71 or K31 peptides were taken up by CD4^+^ and CD8^+^ T cells, leading to IFN-γ expression. C3H/HeJ mice developed AA after 10 weeks of subcutaneous treatment with dendritic cells (DCs) loaded with MHCI- or MHCII-binding K71 or K31 peptides. Soluble peptides are mostly nonimmunogenic and frequently can be immunosuppressive. C3H/HeJ mice were induced to develop AA by AA skin transplantation. After weekly intravenous injection of 100 μg MHCI- or MHCII-binding K71 or K31 peptides, the mean AA affected area was significantly decreased. However, only ~50% mice were prevented from AA initiation or progression. CD4^+^ or CD8^+^ T cells from treated mice were unresponsive to peptide-loaded DCs, as evidenced by reduced trogocytosis. In summary, vaccination with soluble K71 or K31 peptides makes T cells unresponsive to DC-presented K71 and K31 peptides, which significantly retards the induction of AA and prevents its progression in a C3H/HeJ mouse model [101].

The immunomodulatory neuropeptide Substance P (SP) is expressed by sensory nerve fibers, which was increased in the early stage of AA, while decreased in the advanced stage of AA. Treatment with SP in skin affected by AA accelerates HF regression (catagen). Degranulating mast cells and CD8^+^ lymphocytes significantly increased. SP increased the expression of granzyme B in CD8^+^ cells through the neurokinin-1 receptor (NK-1R) in a C3H/HeJ mouse model for AA [102].

Vasoactive intestinal peptide (VIP) is an immunoinhibitory neuropeptide released by perifollicular sensory nerve fibers. The immunoinhibitory effect is mediated by binding to its receptors. VIP receptors (VPAC1, VPAC2) are expressed in hHFs, whose expression levels are reduced in AA. VIP partially prevented the collapse of immune privilege in *ex vivo* hHFs induced by IFN-γ. However, the function was mild in already collapsed hHFs. An amount of 10^−9^ M VIP achieved the best protective effect [103].

Food-derived immunostimulatory peptides also play a role in hair regeneration. Gly-Leu-Phe (GLF), an immunostimulating peptide derived from α-lactalbumin, has demonstrated the ability to prevent alopecia induced by the anticancer agent etoposide in a neonatal rat model. The function was inhibited by pyrilamine, a histamine H_1_ receptor antagonist, indicating that the anti-alopecia effect is mediated by histamine release [104].

Soymetide-4 (MITL), an immunostimulating peptide derived from the soybean β-conglycinin α’ subunit, suppressed alopecia induced by etoposide in neonatal rats. The function can’t be inhibited by pyrilamine and cimetidine, but is inhibited by indomethacin, a cyclooxygenase inhibitor, AH-23848B, an antagonist of the EP_4_ receptor subtype, and PDTC, an inhibitor of NF-κB. It was hypothesized that MITL stimulates cyclooxygenase, which promotes the production of PGE_2_ to activate NF-κB, inhibiting the HF apoptosis [105,106].

Synthetic anti-inflammatory peptides also exist. For instance, TAT-GILZ is a synthetic peptide derived from the glucocorticoid-induced leucine zipper (GILZ) fused to a TAT sequence. It accelerated hair growth in BALB/c and nude male mice. Immunofluorescence showed increased co-expression of Lhx2 and CD133, the indicator of functional HFSCs. Osteopontin and CD44, markers of the HFSC niche, were also increased. The majority of FOXP3 is expressed in regulatory T cells (Tregs). The co-expression of FOXP3 and glucocorticoid receptor (GR) was increased after TAT-GILZ treatment. According to previous studies, GR expression is correlated with Treg’s function for HFSC activation, local homeostasis, and hair growth. Importantly, TAT-GILZ induced more mature, larger HFs than 5% finasteride or minoxidil in BALB/c male mice [107].

### 3.4. ECM Remodeling and Structural Reinforcement Peptides

The extracellular matrix (ECM) surrounding the DP provides structural support and biochemical signals essential for follicle maintenance and cycling. ECM-targeting peptides reinforce follicular architecture and promote the synthesis of structural proteins (Figure 3D).

Acetyl tetrapeptide-3 (Ac-KGHK) is a synthetic biomimetic peptide designed from a signal peptide, which is a short bioactive fragment released from ECM proteins. Immunofluorescence showed that Ac-KGHK significantly stimulated the production of collagen III and laminin in human fibroblasts (MRC5). The upregulation of collagen VII was confirmed in human skin explants by immunohistological staining. The mixture of red clover extract and Ac-KGHK inhibited the production of IL-8 in NHDF cells induced by IL-1α, which was more efficient than using red clover extract alone. The function of the mixture was further confirmed in a clinical study. Two groups of patients (30 volunteers) were treated with a placebo or the mixture. After 4 months, the anagen/telogen hair ratio was significantly higher in the treated group than in the placebo group [25,108,109]. Co-delivery of Ac-KGHK and myristoyl pentapeptide-4 by nanoliposomes (CAM-NLPs) can promote the proliferation of HaCaT cells and hDPCs.

Similarly, targeting the hair shaft’s structural components, CAM-NLPs were reported to stimulate the expression of collagen III and epidermal keratins in HaCaT cells [109]. It increased the hair regrowth in the testosterone-induced AGA mouse model.

In addition, the naturally occurring tetrapeptide acetyl-N-Ser-Asp-Lys-Pro (AcSDKP) was recognized as a potent angiogenic factor. It stimulated the proliferation of human HaCaT cells and dermal NHDF fibroblasts. An *in vitro* clonogenic assay revealed that AcSDKP increased the number of colonies formed by human keratinocyte stem cells (KSC) or keratinocyte progenitors, albeit slightly and significantly. It also promoted the proliferation of hDPCs *in vitro* and the elongation in *ex vivo*-cultured HFs. Collagen IV, laminin 5, and keratin 19 were found to be increased in HFs after being treated with 10^−10^ M AcSDKP [110]. AcSDKP increased the expression of collagen I in human dermal fibroblasts and the expression of tight junction proteins in human keratinocytes. IF and WB validated the upregulation of SIRT1 in human keratinocytes and fibroblasts.

Furthermore, collagen hydrolysate (CH) extracted from Mozambique tilapia (*Oreochromis mossambicus*) has been found to promote wound healing and cell proliferation. H_2_O_2_ decreased catalase activity in hDPCs, an effect restored by CH treatment. CH can increase the mRNA levels of hair-related cytokines, such as IGF-1, VEGF, and TGF-β1, in hDPCs, while decreasing the mRNA levels of inflammatory cytokines, such as TNF-α and IL-1β. In the C57BL/6 mouse model, 1000 mg/kg CH shows a better hair regrowth effect than 1 mg/kg finasteride [90,111,112]. RT-qPCR of mouse dorsal skin after treatment further confirmed the upregulation of IGF-1, VEGF, Elastin, and HAS2, while the downregulation of TNF-α and IL-1β. Elastin and HAS2 belong to ECM production-related factors.

### 3.5. Autophagy, Metabolic, and Other Emerging Pathways

Several peptides act through less conventional mechanisms, including regulation of autophagy, activation of metabolic receptors, and other mechanisms that remain to be fully elucidated (Figure 3E).

The AC2 peptide was isolated from the *Trapa japonica* fruit. WST-1 assay showed no cytotoxicity from AC2 peptide in hDPCs. An amount of 10 mg/mL AC2 can even rescue the proliferation defects caused by 1 mg/mL DHT. WB showed increased Cyclin-E1 in hDPCs after AC2 treatment. The mechanistic study indicated that AC2 activates mTORC1 signaling, thereby suppressing autophagy and apoptosis. WB showed increased p-mTOR, Raptor, p-4E-BP1, p-S6, Bcl-2 and p62, while decreased Bax, Apaf-1, cleaved caspase-3, Beclin, and LC3-I/II. The decreased autophagy and apoptosis were further confirmed by flow cytometry. To validate the function derived from the AC2 peptide, the synthesized AC2 showed a similar effect in the WST-1 assay compared to the extracted AC2 peptide [113].

Tat-BECN1 is derived from the autophagy-associated beclin1 protein linked to the TAT protein derived from the HIV virus. It can block GLIPR2-mediated inhibition of BECN1, thereby inducing net autophagy. Autophagic activity is blocked in HFs of C3H/HeJ AA mice. The autophagy inhibitor chloroquine accelerated disease onset in AA mice. However, Tat-BECN1 treatment delayed the onset of AA in the C3H/HeJ mouse model [114]. The area of hair loss was significantly decreased. Importantly, T cells and macrophages didn’t change in the skin and the skin-draining lymph nodes. This suggests that autophagy plays a direct role in regulating hair regeneration.

Metabolic receptors may also be targeted. A small transdermally deliverable peptide, P5 (GLYYF), was designed to bind adiponectin receptor 1 (AdipoR1). GST pulldown confirmed the interaction. P5 activated the AMPK signaling pathway in ORS cells and DPCs in a dose-dependent manner. AdipoR1 knockdown by siRNA will reduce the effect, which means the function of P5 relies on AdipoR1. P5 also increased mRNA and protein levels of hair growth factors in DPCs, including IGF-1, VEGF, HGF, PDGFA, and FGF7. In *ex vivo* hHF organ culture model, 25 μM P5 treatment significantly promoted hair shaft growth. Ki67 and pAMPK were found to increase according to IF. Topical P5 treatment induced hair regrowth in C57BL/6 mice. An amount of 0.1 mM P5 achieved an effect comparable to 3% minoxidil [115]. Anagen induction score was significantly increased. In *Adipoq^−/−^* mice, P5 treatment can achieve similar effects. However, in *Adipor1^−/−^* mice, the function of P5 was completely abolished, further supporting the notion that P5 function relies on AdipoR1. This peptide is notable for its ability to be delivered transdermally into human skin, addressing a key challenge in peptide therapy. Molecular docking was used to analyze the binding of P5 and AdipoR1. Mutations and GST pulldown were used to validate the model, which serves as a good example of rational design.

Ultra-high molecular weight γ-PGA (UHMW γ-PGA) is an unusual anionic polypeptide. It inhibited 5α-reductase activity *in vitro* in a dose-dependent manner. It promoted hair regrowth by effectively inducing the anagen phase in telogenic C57BL/6 mice. However, 30 mg/mL UHMW γ-PGA can’t keep up with the hair growth rate of 5% minoxidil [116].

The parathyroid hormone-related peptide (PTHrP) regulates skin angiogenesis and reverses delayed catagen in the TSP1-KO mice [117]. A new model proposes that it can induce both the transition from anagen to catagen and from telogen to anagen, thereby facilitating the hair cycle. However, the dispute over the effects of PTHrP agonists and antagonists in hair loss remains unresolved [118].

Additionally, a pentapeptide, Gly-Pro-Ile-Gly-Ser (GPIGS), was originally identified in conditioned medium from *Bacillus* sp. M18 cultures. It was reported that GPIGS promoted the proliferation of human hair keratinocytes *in vitro* and the elongation of hair shafts in hHF organ cultures *ex vivo*. The function was further confirmed in a clinical study of Japanese men with AGA [119]. GPIGS increased the baldness grade after 4 months of treatment. However, the increase in hair density was not significant. No causal adverse effects were noted.

## 4. The Application Challenges of Short Peptides

### 4.1. Peptides’ Self-Defects

As discussed in the previous chapter, peptides offer several key advantages, including high specificity, favorable biocompatibility, and the potential for combination therapies. However, their clinical application is frequently hindered by inherent physicochemical instability. Peptides are highly susceptible to enzymatic degradation and hydrolysis because they are composed primarily of natural L-amino acids and lack robust secondary or tertiary structures. Consequently, structural modifications are necessary to enhance the stability and pharmacokinetic properties of peptides [120]. Nevertheless, these modifications may introduce further complexities in dosage standardization during clinical translation.

Furthermore, peptides typically exhibit poor membrane permeability [121]. Although new peptides are developed rapidly, the application of corresponding delivery systems has lagged. Many studies rely on oral or injection administration, which present significant translation barriers. Injections will reduce patient compliance, while oral administration struggles with off-target effects. Given that most peptides modulate specific signaling pathways, it is challenging to restrict peptides’ pharmacological activity to the scalp. At present, peptides that have advanced to Phase I clinical trials, such as acetyl tetrapeptide-3, GHK, and GPIGS, are delivered topically in lotions or creams [18,25,119]. Consequently, the integration of advanced nanotechnologies, including microneedles, nanoliposomes, and nanostructured lipid carriers, appears critical for successful clinical translation [70,109,122].

Safety, particularly regarding immunogenicity, constitutes another critical challenge. Current studies are predominantly derived from *in vitro* hDPCs and ORS cells, as well as *in vivo* murine models [95,115]. Occasionally, *ex vivo* human skin cultures are involved. In murine studies, peptide administration is conventionally timed to the telogen phase, and minoxidil is often used as a positive control [123]. Functional assessments are widely implemented, while immunogenic profiling remains sparse. Although peptides have been shown to exhibit favorable biocompatibility, their immunogenicity must be rigorously evaluated in humans. Since T lymphocytes are highly specialized in recognizing peptide antigens, therapeutic peptides inherently possess the potential to act as potent immune triggers. Both the US Food and Drug Administration (FDA) and the European Medicines Agency (EMA) have issued strict guidelines for immunogenicity assessment, which are highly relevant to the development of peptide-based alopecia treatments [124]. Finally, the peptide manufacturing process is intrinsically linked to immunogenic risk. Beyond commercial feasibility, manufacturing-derived impurities can independently provoke adverse immune responses.

### 4.2. Pathway Convergence and Pleiotropic Effects

Studies based on pathway classification indicate that many peptides can activate multiple signaling cascades simultaneously (Table 4). For example, APCP can activate both the Wnt/β-catenin and PKA/AKT/ERK pathways. GHK-Cu can regulate VEGF, ECM, and inflammatory pathways. TAT-GILZ exhibits both anti-inflammatory effects and functions to maintain the stem cell microenvironment. AIMP1, secreted from HFSCs, activates DPCs and follicular stem cells by modulating the β-catenin, AKT, and ERK pathways. This multi-pathway activation aligns with the concept of multi-effect therapy [20,21,89,90,96,107]. Unlike the limited hormonal action of finasteride and androgen receptor antagonists, peptides provide a broader biological intervention. This “multi-hit” regenerative effect positions peptides as potent inducers of hair follicle revitalization.

However, it is worth noting that there remains a lack of direct experimental evidence to demonstrate the synergistic effects of these pathways in the treatment of hair loss. The superiority of multi-target peptides over single-target peptides has not been systematically evaluated. Future research should focus on using pathway inhibitors and combination peptide therapies to determine true synergy and the independent effects of these treatments.

From a translational medicine perspective, understanding the mechanisms of peptide pathway interactions can facilitate the development of rational drug combination strategies. For example, combining Wnt-activating peptides with angiogenic peptides could simultaneously regulate the hair follicle cycle and nutritional supply. While this mechanism-based combination strategy shows promising application potential, it requires clinical validation.

Peptides can be co-administered with agents such as plant extracts and vitamins, which further protect the scalp and repair the hair shaft [125]. Meanwhile, peptides can act as delivery vehicles that localize and potentiate conventional drugs at the scalp surface, as suggested by the carrier-free finasteride-peptide system [126]. This enables highly personalized “stacked” protocols. For example, a single topical product targets androgenic, inflammatory, microcirculatory, and structural drivers of hair loss in parallel.

**Table 4:** Summary of peptides for hair loss treatment.

Classification	Peptide	Hair Loss Type	Evidence Types	Experimental Model	Delivery Approach	Dosage ^1^ (If Noted)	Primary Readout Metrics [123]	Mechanism	Credibility	Amino Acid Sequence	References
Collagen-based natural peptides	Recombinant human type XVII collagen (rhCOL17A1)	AGA ^2^	Experimental evidence	TES-treated C57BL/6 mice	Topical administration	0.02%, 0.1%, or 0.5% *w*/*v*	Hair coverage	Activation of Wnt/β-catenin and SHH/GLI signaling pathways.	*In vivo*	Patent CN118373900	[92]
	AP collagen peptides (APCPs)	-	Experimental evidence	C57BL/6 mice	Injection	300, 600, or 900 mg/kg	Hair regrowth area	Increased expression of β-catenin and VEGF ^2^.	*In vivo*	3% Gly-Pro-Hyp	[89,90]
	Low molecular weight collagen peptide (LMWCP)	-	Experimental evidence	C57BL/6 mice	Oral administration	615 mg/kg or 820 mg/kg	Hair regrowth area	Increased expression of Type I and II hair keratins, β-catenin, and VEGF.	*In vivo*	3% Gly-Pro-Hyp and 15% tripeptide	[91]
	Collagen hydrolysate (CH)	-	Experimental evidence	C57BL/6 mice	Oral administration	500 mg/kg or 1000 mg/kg	Hair regrowth index	Reduces oxidative stress, upregulates Wnt/β-catenin, and downregulates BMP pathways	*In vivo*	High levels of Pro-Hyp dipeptide	[111,112]
Metal-binding peptides	GHK-Cu	AGA	Clinical evidence	Male AGA patients	Topical spray	-	Hair count	Remodels ECM, stimulates dermal fibroblast, increases VEGF expression, and decreases DPC ^2^ apoptosis.	Phase I clinical data	Gly-His-Lys-Cu^2+^	[18,20,21,127]
	AHK-Cu	-	Experimental evidence	Human hair follicle organ	-	10^−13^ M to 10^−7^ M	Hair follicle elongation	Stimulates DPC proliferation and prevents apoptosis.	*Ex vivo*	L-Ala-L-His-L-Lys-Cu^2+^	[23]
Immunomodulatory peptides	Soluble K71 or K31 peptides	AA ^2^	Experimental evidence	C3H/HeJ mice	Vaccination with soluble K71 or K31	100 μg	Area of hair loss	Induction of long-lasting T-cell anergy to peptide antigens.	*In vivo*	-	[101]
	Substance P (SP)	AA	Experimental evidence	C3H/HeJ mice	Intracutaneous injection	1 mg/mL	Hair cycle	Increases mast cell degranulation and CD8+ lymphocytes, promoting catagen entry.	*In vivo*	Arg-Pro-Lys-Pro-Gln-Gln-Phe-Phe-Gly-Leu-Met-NH_2_	[102]
	GLF	Chemotherapy-induced alopecia	Experimental evidence	Neonatal rat model	Intraperitoneal injection or oral administration	100 mg/kg or 300 mg/kg	Photographs and histology (skin sections)	Histamine release (proposed).	*In vivo*	Gly-Leu-Phe	[104]
	Soymetide-4	Chemotherapy-induced alopecia	Experimental evidence	Neonatal rat model	Oral administration	300 mg/kg	Hair area	PGE_2_-mediated suppression of hair-matrix apoptosis via NF-κB activation.	*In vivo*	MITL	[105]
	VIP	AA	Experimental evidence	Cultured human hair follicles	Bath application in culture	10^−12^, 10^−9^, and 10^−7^ M	MHC I/II and β2-microglobulin expression; MHCII^+^ cell counts	Maintains hair follicle immune privilege.	*Ex vivo*	UniProt P01282	[103]
Other natural peptides	AC2 peptide	AGA	Experimental evidence	Human dermal papilla cells (hDPCs)	-	10 mg/mL	Proliferation, autophagy, and apoptosis	Strengthen mTOR-raptor interaction.	*In vitro*	NMR profile	[113]
	Water-soluble chicken egg yolk peptides	FPHL ^2^	Clinical evidence	FPHL patients	Oral administration	250 mg/day	Hair density	Stimulates VEGF production	Phase I clinical data	-	[100]
	Low molecular weight porcine placenta peptide (Placenderm^®^)	AGA	Experimental evidence	C57BL/6 mice	Oral gavage	50, 100, or 200 mg/kg	Skin color score, hair thickness, and hair follicle regeneration	Modulation of Wnt/β-catenin signaling.	*In vivo*	-	[88]
	CyRL-QN15	Type 2 diabetes	Experimental evidence	Kunming and C57BL/6 mice	Topical administration	100 nmol/L	Hair regeneration area, hair length, and hair cycle	Binding to the Frizzled-7 receptor to upregulate β-catenin and Cyclin D1	*In vivo*	CQFHYMC	[93]
Biomimetic peptides	Ac-KGHK	AGA	Clinical evidence	AGA patients	Topical administration	1, 10, or 100 μM	Hair mass index	Stimulates DP ECM ^2^ protein production and reduces pro-inflammatory cytokines.	Phase I clinical data	Acetyl-Lys-Gly-His-Lys	[25,108]
	TN41	-	Experimental evidence	C57BL/6 mice	Topical administration	100 nM	Hair regrowth area	Activates AKT and ERK pathways, increases β-catenin, and enhances DPC activation.	*In vivo* and *ex vivo*	Amino acids 6–46 of AIMP1	[96]
	TAT-GILZ	-	Experimental evidence	Male BALB/c and nude (athymic, nu/J) mice	Intradermal injection	20 μL of 2 μg solution	Hair growth area	Increases counter-inflammatory signaling.	*In vivo*	[128]	[107]
	PRG-RADA16	-	Experimental evidence	C57BL/6 and BALB/c nu/nu mice	Transplantation	1% *w*/*v*	Number of hair shafts per wound	Promotes proliferation of skin-derived precursors.	*De novo*	Ac-RADARADARADARADAGPRGDSGYRGDS-CONH_2_ and Ac-RADARADARADARADA-CONH_2_	[97]
	Lgr5-binding octapeptide	AGA	Experimental evidence	Human hair cells	-	10 μM, 50 μM, or 100 μM	Proliferation and differentiation	Activation of Wnt/β-catenin signaling via Lgr5 targeting.	*In vitro*	NH_2_-LKRYKHLV-OH	[95]
	GPIGS	AGA	Clinical evidence	22 Japanese men	Topical lotion	0.1% *w*/*v*	Hair diameter and density	Stimulates the proliferation of hair keratinocytes.	Phase I clinical data	Gly-Pro-Ile-Gly-Ser	[119]
	Sh-Polypeptide 9	AGA	Experimental evidence	hDPCs ^2^ and microvascular endothelial cells	-	2.5 mg/mL, 5 mg/mL, or 10 mg/mL	Tubulogenesis, cell viability, and proliferation	Promotes endothelial tubulogenesis, VEGF production, and increases β-catenin in hDPCs.	*In vitro*	-	[99]
	PTHrP	AA and chemotherapy-induced alopecia	Experimental evidence	SKH-1 hairless, K14-PTHrP, FGF5-KO, and TSP1-KO mice	Agonists or antagonists	-	Hair length and hair cycle	Promotes the anagen-to-catagen transition by inhibiting angiogenesis.	*In vivo*	UniProt P12272	[117,118,129]
	AcSDKP	-	Experimental evidence	Cultured hair follicles	-	10^−11^–10^−7^ M	Hair length	Increases the proliferation of human keratinocytes, fibroblasts, and hDPCs.	*Ex vivo*	Acetyl-N-Ser-Asp-Lys-Pro	[110]
	P5	-	Experimental evidence	*Adipoq*^−/−^, *Adipor1*^−/−^, and C57BL/6 female mice	Topical administration	0.1 mM	Hair cycle score	Activates adiponectin receptor 1.	*In vivo*	GLYYF	[115]
	UHMW γ-PGA	AGA	Experimental evidence	Telogenic C57BL/6 mice	Topical administration	30 mg/mL	Skin color and number of HFs	Inhibits 5α reductase.	*In vivo*	Poly-γ-Glutamic acid	[116]
	Myristoyl pentapeptide-4	AGA	Experimental evidence	C57BL/6 mice	Nanoliposome topical co-delivery	3% (*w*/*w*)	Time to skin pigmentation/blackening and hair growth	Upregulates VEGF and β-catenin expression.	*In vivo*	KTTKS	[109]
	PTD-DBM peptide	AGA	Experimental evidence	C3H mice	Topical administration	2 mM	Hair weight and HF number	Blocks CXXC5-Dvl interaction to active Wnt/β-catenin signaling.	*In vivo*	RRRRRRRRGGGGRKTGHQICKFRKCK-FITC	[94]
	FOL-005	AGA	Experimental evidence	C57BL/6 mice	Microparticle topical formulation	0.01, 0.1, and 0.5% (*w*/*w*)	Hair growth score	Targets specific follicular cell layers.	*In vivo*	VDTYDGDISVVYGLR	[122]
	RK81^QTY^	-	Experimental evidence	Female C57BL/6 mice	Microneedle	100 mg/mL, 100 μL	Hair weight, thickness, and length. HF’s number and cycle	Upregulating the PI3K/AKT/Nf−κB signaling axis	*In vivo*	-	[130]

^1^ A more comprehensive list of dosages is provided in Table A1. ^2^ Abbreviations: AGA, androgenetic alopecia; AA, alopecia areata; DP, dermal papilla; DPC, dermal papilla cell; hDPCs, human dermal papilla cells; ECM, extracellular matrix; FPHL, female pattern hair loss; HF, hair follicle; VEGF, vascular endothelial growth factor.

### 4.3. Research Limitations

To our surprise, most studies have not explained how they determined the optimal dosage. Low dosages cannot achieve ideal effects. High dosages may increase the risk of side effects and raise the cost of peptides. Peptides such as Placenderm^®^, APCP, LMWCP, AHK-Cu, and AC2 have been tested *in vitro* for cell viability [23,88,89,91,113]. Additionally, the safety of RK81^QTY^ was further evaluated *in vivo* [130].

For peptide design, natural discovery remains the primary source. Rational design is not widely used, except for P5, PRG-RADA16, and RK81^QTY^ [97,130]. However, as peptide structure and binding affinity prediction advance, high-throughput virtual screening should be employed to reduce development time. Meanwhile, mechanistic studies, especially those on hair cycle regulation, are the cornerstone.

Currently, peptides are primarily incorporated into lotions and formulations as additives, such as QR678 Neo^®^, Placenderm^®^, and Capixyl^TM^ [88,108,131,132]. Aside from PP405, which is undergoing a Phase II clinical study, none of these peptides is classified as a medicine [10]. One reason for this is that the application of cosmetic ingredients is much simpler than that of pharmaceuticals, which require costly clinical trials. Pharmaceuticals also require high-purity peptides, which raise production standards. Another factor may be the heterogeneity of patient responses.

Despite advancements in peptide research, several limitations remain. Firstly, there is a notable lack of robust animal models to study the underlying mechanisms of various types of hair loss. For instance, in the studies of APCP, LMWCP, CH, TN41, and RK81^QTY^, simply shaving the dorsal hair of mice does not accurately replicate any disease, as the niche and hair follicles themselves remain healthy [89,91,96,111,130]. Promoting hair growth in these healthy follicles does not necessarily translate to effective treatments for all forms of hair loss. Similarly, in studies of AC2, the Lgr5-binding octapeptide, Sh-Polypeptide 9, and AcSDKP, which promote the proliferation of various cell types, including DPCs, HFSCs, and matrix cells, do not demonstrate therapeutic effects *in vivo* [95,99,110,113].

Secondly, there is a lack of standardized methods for assessing treatment effects (Table 4). Different studies employ varying criteria to define promotion effects, such as cell proliferation, mobility, DNA synthesis, hair counts, length, density, thickness, and growth rate. The most critical measure is whether the treatment enables normal hair growth in bald areas. However, establishing objective, standardized evaluation criteria requires careful consideration.

Thirdly, the clinical studies of GPIGS and Ac-KGHK lack sufficient numbers of participants. Additionally, all clinical studies, including those on GPIGS, Ac-KGHK, GHK-Cu, and water-soluble chicken egg yolk peptides, do not provide meaningful comparisons with established drugs such as minoxidil or finasteride, which undermines their credibility [18,25,100,119].

In summary, the clinical translation of peptide therapeutics for alopecia requires overcoming substantial hurdles related to physicochemical stability, permeability, specificity, immunogenicity, and regulatory compliance. Despite these challenges, peptides remain highly promising candidates for the development of targeted, effective, and next-generation hair-loss treatments.

## 5. Perspectives

### 5.1. Nanotechnologies Are Employed to Overcome the Challenges Associated with the Delivery of Peptides

Intradermal injection requires professional assistance. Meanwhile, the patient’s compliance and treatment cycle will be affected. To address challenges such as inadequate permeability, diminished bioavailability, and regulatory considerations of peptides, nanotechnologies have been employed, including microneedles and liposomes. Research indicates that 200 nm particles can reach the isthmus of hair follicles, while 20–40 nm particles can access the bulb [133].

For instance, RK81^QTY^ is a rationally designed keratin developed using QTY code methodology to enhance water solubility and fabricate microneedles, exhibiting therapeutic effects comparable to minoxidil in C57BL/6 mice. It was found that RK81^QTY^ improved angiogenesis and activated the PI3K/AKT/Nf−κB pathway. The biocompatibility of microneedles assessed by the CCK-8 assay was favorable [130].

Globefish skin collagen peptides with dissolving microneedles (GSCPs-MNs) had a similar effect to promote hair regrowth in AGA mice compared with minoxidil. In contrast, the effect of topical GSCPs was similar to that of the control. GSCPs-MNs promoted cell proliferation and collagen synthesis, downregulated the secretion of the inflammatory factors TNF-α and IL-1β, and ultimately improved vascularization around HFs. Microneedles that generate skin micro-wounds have an independent function in improving hair growth [134].

Nanoliposomes loaded with copper peptide, acetyl tetrapeptide-3, and myristoyl pentapeptide-4 (CAM-NLPs) had a particle size of 40 nm, promoting the proliferation and migration of hDPCs *in vitro*, which improved hair growth by upregulating the protein levels of VEGF and β-catenin while downregulating TGF-β1 in the skin of testosterone-treated C57BL/6 mice *in vivo*. While the drug release of CAM-NLPs was slower than that of free peptides, the penetration and cellular uptake were both higher in the CAM-NLPs group [109].

Although these technologies are still in the experimental stage and lack sufficient clinical data, nanosystems represent promising strategies for delivering peptides [133].

### 5.2. Peptides Are Used as an Assistant to Improve the Treatment Effectiveness

Regarding different peptide mechanisms, their collocation can achieve synergistic effects. Given the high cost of peptide production and the limited availability of existing therapies, some researchers also use peptides as adjuncts, combining them with traditional medicines, plant extracts, or physical therapies to improve treatment efficiency.

One notable example is the QR678 Neo^®^ formulation, which contains Sh-polypeptides and copper tripeptide. Cytotoxicity studies have shown that each factor is safe for human keratinocytes and fibroblasts [131]. Intradermal administration of QR678 Neo^®^ increased mean hair counts (5.64) in 20 female patients with TE [132]. Additionally, QR678 Neo^®^ can serve as a storage solution to assist hair transplant surgeries, improving the terminal hair count (181.02 vs. 150.45) [135].

Biosea^®^ Revive Serum (BRS), which contains biotinoyl tripeptide-1 and *Phyllanthus emblica* fruit extract, is a hair care product that has been demonstrated to increase hair density (107.2%) in male hair loss patients when sprayed twice daily. A mechanistic study showed that 1.25% BRS increased proliferation and reduced reactive oxygen species (ROS) generation and 5α-reductase expression in hDPCs *in vitro*, findings comparable to those of minoxidil [136].

There are also examples of peptides combined with physical therapies. A non-ablative erbium glass fractional laser, followed by the application of 0.05% topical finasteride and growth factors (including bFGF, IGF, VEGF, and 1% copper peptide), was applied to treat four AGA patients, resulting in improved hair regrowth and density. Fractional lasers were believed to assist in penetration of topical medications, and the micro-wounding would further activate HFs [137]. Another example was carried out in type III to IV male AGA patients aged 28–55 years over five months. Topical administration of 0.5% minoxidil, 0.1% dutasteride, and 1.2% copper peptide via tattooing (MDCT) achieved an increased median top scalp area regrowth (TSAR, 26.5% vs. 10%) compared with 3-monthly sessions of minoxidil-dutasteride tattooing (MDT) [138].

Peptides can be used as the main component or as a supplement in any solution, depending on the requirements.

### 5.3. Potential Targets for Designing Peptides to Treat Hair Loss

Beyond directly activating the Wnt signaling pathway, there is a need to explore new peptides. Based on advanced physiological studies, some new targets could be tested.

Research into congenital generalized hypertrichosis terminalis (CGHT) has revealed that the potassium channel KCNJ2 in dermal fibroblasts mediates membrane hyperpolarization and enhances fibroblast Wnt signaling, promoting hair growth [139]. Biomimetic agonist peptides can be developed to activate KCNJ2 specifically.

GPR30, also known as G protein-coupled estrogen receptor 1, is a membrane-associated receptor that mediates intracellular signaling cascades. Activation of GPR30 using the selective agonist G-1 has been shown to promote hair growth in C57BL/6J mouse skin by upregulating Wnt and Hedgehog signaling pathways [140]. Future research could focus on rationally designing agonist peptides for GPR30.

Thrombospondin type 1 domain containing 4 (THSD4) is an extracellular matrix (ECM) protein located at the interface of the DP and hair matrix (HM), which is significantly downregulated in aged hair follicles. THSD4 has been shown to promote hair growth by enhancing the interaction between DP and epithelial cells via the SDC4-THSD4-CXCL1 signaling axis [141], underscoring the concept that improving the interaction between DP and HM can promote hair regrowth. One type of peptide can be designed based on this mechanism.

The pathogenesis of female-pattern hair loss (FPHL) remains poorly understood. Studies have indicated that luteinizing hormone (LH) levels and LH receptor expression increase in premenopausal patients with FPHL. Activation of LH receptors leads to calcium influx mediated by transient receptor potential canonical (TRPC) channels. Daily subcutaneous injections of LH in female C57BL/6J mice led to hair loss during the telogen phase, whereas TRPC inhibitors mitigated this effect. The LH/LHR/TRPC axis is a novel contributor to FPHL, and TRPC inhibition presents a promising therapeutic strategy. However, TRPC inhibitors may decrease plasma LH levels, affecting other organs. Therefore, specific topical inhibitory peptides could be designed to selectively inhibit LH receptors in hair follicles to treat FPHL [142,143].

Safety, effectiveness, specificity, and quality control are important factors for further peptide design.

## 6. Conclusions

Despite the multitude of patented and advertised “anti-hair loss” agents, effective treatment remains more of an expectation than a reality in this field [144]. Peptides exhibit high biocompatibility, various mechanisms, rich sources, and synergistic functions. However, the high production costs, low permeability, insufficient clinical trials, and lack of comparison with standard medicines hinder the commercialization of most peptides. In conclusion, while there is still a long way to go in the quest to cure hair loss, significant progress has been made. Peptides combined with nanotechnology are advancing and hold potential for future applications in hair loss treatment.

## Figures and Tables

**Table 1:** Summary of hair loss-related diseases.

Etiology	Common Agents	Diseases	References
Hormonal imbalances	Androgen	Androgenic Alopecia	[39]
	Thyroid	Hypothyroidism	[40]
		Hyperthyroidism	[41]
Autoimmune disorders		Alopecia Areata	[42]
		Lupus Erythematous	[43]
Infection	Dermatophytes	Tinea capitis	[7]
	*Corynebacterium* spp.	Trichobacteriosis	[44]
	*Treponema pallidum*	Syphilis	[45]
	*Staphylococcus aureus*	Folliculitis	[46]
Stress-induced	Physical or mental stress	Telogen Effluvium	[8]
	Radio or chemotherapy	Anagen Effluvium	[47]
	Physical tension	Traction Alopecia	[48]

**Table 2:** Summary of present medications.

Drug	Mechanism	Effects ^1^	Typical Onset	Primary Side Effects	Deficiencies	References
Minoxidil	Vasodilation and prolongation of anagen	~60% response rate at 1 year	16–52 weeks	Local irritation (reported 3.9–46.5%); irritant contact dermatitis, scaling, pruritus; hypertrichosis	Local tolerability issues; variable response between individuals	[29,30,32]
Dutasteride	Inhibiting type I and II 5α-reductase	Up to +22.04 hairs/cm^2^ after 24 weeks	4–12 weeks	Sexual dysfunction	Possible increased risk of high-grade prostate cancer; potential for mood effects (including depression); systemic sexual adverse effects similar to finasteride	[55,56,57,58,59,60,61,62,63,64,65]
Finasteride	Inhibiting type II 5α-reductase	Up to +5.88 hairs/cm^2^ after 24 weeks	24–48 weeks	Sexual dysfunction; systemic effects	Contraindications similar to dutasteride	[55,56,57,58,59,60,61,62,63,64,65]
Pyrilutamide (Phase III)	Androgen receptor antagonist	Up to +15.34 hairs/cm^2^ (males)	12–24 weeks	Mild or no significant adverse events reported in trials to date	Limited clinical data	[66]
ET-02 (Phase II)	Androgen modulation	~6-fold increase compared with placebo	5–8 weeks	Low adverse event rates reported	Limited clinical data	[67]
PP405 (Phase II)	Peptide-mediated dormant follicle stem cell activation	31% of participants achieved >20% hair density increase in 8 weeks	4–8 weeks	Minimal; primarily local reactions reported	Insufficient information on long-term efficacy and safety	[10]

^1^ Clinical evidence is exhibited.

## Data Availability

No new data were created or analyzed in this study. Data sharing is not applicable to this article.

## Abbreviations

The following abbreviations are used in this manuscript:

AA	Alopecia areata
AR	Androgen receptor
AGA	Androgenetic alopecia
APCP	AP Collagen Peptide
5ARIs	5α-reductase inhibitors
CH	Collagen hydrolysate
DP	Dermal papilla
DPCs	Dermal papilla cells
DHT	Dihydrotestosterone
DCs	Dendritic cells
ECM	Extracellular matrix
FGF	Fibroblast growth factor
FPHL	Female-pattern hair loss
HFs	Hair follicles
hHFs	Human hair follicles
HFSCs	Hair follicle stem cells
GSCPs	Globefish skin collagen peptides
GSCPs-MNs	Globefish skin collagen peptides with dissolving microneedles
hDPCs	Human dermal papilla cells
HM	Hair matrix
IF	Immunofluorescence
LH	Luteinizing hormone
LLLT	Low-level laser therapy
LMWCP	Low-molecular-weight collagen peptides
PRP	Platelet-Rich Plasm
PTHrP	Parathyroid hormone-related peptide
SKPs	Skin-derived precursors
Tregs	Regulatory T cells
TE	Telogen effluvium
TGF-β	Transforming growth factor-β
TCM	Traditional Chinese medicine
THSD4	Thrombospondin type 1 domain containing 4
VIP	Vasoactive intestinal peptide
VEGF	Vascular endothelial growth factor
WB	Western blot

## Appendix A

**Table A1:** Dosage and duration of peptide treatment.

Peptide	Evidence Types	Optimal Dosage	Duration
Placenderm^®^	Experimental evidence	200 mg/kg body weight	Five times per week for 23 days
APCP	Experimental evidence	900 mg/kg body weight	Seven times per week for 16 days
LMWCP	Experimental evidence	615 mg/kg body weight	Once a day for two weeks
rhCOL17A1	Experimental evidence	0.5% *w*/*v*	Once a day for two weeks
CyRL-QN15	Experimental evidence	100 nmol/L, 20 μL each time	Twice daily for 28 days
Lgr5-binding octapeptide	Experimental evidence	100 μM	72 h
TN41	Experimental evidence	100 nM	Once a day for 10 days
PRG-RADA16	Experimental evidence	1% (*w*/*v*) PRG-RADA16 was diluted with dH_2_O to a working concentration of 0.1% and 0.01% (*w*/*v*)	
AHK-Cu	Experimental evidence	10^−9^ M	
Sh-Polypeptide 9	Experimental evidence	2.5 mg/mL	
Water-soluble chicken egg yolk peptides	Experimental evidence	0.1% *w*/*w* diet, 100 mg/kg	17 days
Soluble K71 or K31 peptides	Experimental evidence	100 μg	Weakly
VIP	Experimental evidence	10^−9^ M	
GLF	Experimental evidence	300 mg/kg body weight	6 days
MITL	Experimental evidence	300 mg/kg body weight	8 days
TAT-GILZ	Experimental evidence	20 μL of 2 μg	Twice at one-week intervals
Ac-KGHK	Clinical evidence	100 μM	
Myristoyl pentapeptide-4	Experimental evidence	40 μM	
GPIGS	Clinical evidence	3 mL lotion containing 0.1% (*w*/*v*) peptide	Twice daily for 4 months
AcSDKP	Experimental evidence	10^−5^ M	6 days
Collagen hydrolysate	Experimental evidence	1000 mg/kg body weight	21 days
AC2	Experimental evidence	10 mg/mL	
Tat-BECN1	Experimental evidence	15 mg/kg body weight	3 times per week for a maximum of 8 weeks
GLYYF	Experimental evidence	0.1 mM	Once a day for 35 days
UHMW γ-PGA	Experimental evidence	30 mg/mL, 150 μL each time	4 weeks
